# Exercise intervention decreases acute and late doxorubicin‐induced cardiotoxicity

**DOI:** 10.1002/cam4.4283

**Published:** 2021-09-15

**Authors:** Fei Wang, Joya Chandra, Eugenie S. Kleinerman

**Affiliations:** ^1^ Division of Pediatrics The University of Texas MD Anderson Cancer Center Houston Texas USA

**Keywords:** cardiotoxicity, doxorubicin, exercise, long‐term survival, myocardial infarction

## Abstract

**Background:**

Doxorubicin (Dox) is one of the most effective chemotherapy agents used to treat adolescent and young adult sarcoma patients. Unfortunately, Dox causes cardiotoxicities that compromise long‐term survival. We investigated whether exercise prevented cardiotoxicity and increased survival following myocardial infarction.

**Methods:**

Juvenile mice received Dox, Dox + exercise (Exer), Dox then exercise or were exercised during and after Dox. Mice were evaluated by echocardiography and histology immediately after therapy and 12 weeks later. Mice subjected to permanent ligation of the left anterior descending artery 90 days after therapy were assessed for survival at 45 and 100 days.

**Results:**

Mice treated with Dox, but not Dox + Exer, had decreased ejection fraction (EF) and fractional shortening (FS) immediately after Dox therapy, which continued to deteriorate over 12 weeks with the development of diastolic failure and fibrosis. Acute Dox‐induced cardiotoxicity was documented by induction of autophagy and abnormal mitochondria and vascular architecture with decreased pericytes. These abnormalities persisted 12 weeks after therapy. These acute and late changes were not seen in the Dox + Exer group. Initiating exercise after Dox therapy promoted recovery of EF and FS with no functional or histologic evidence of Dox‐induced damage 12 weeks after therapy. Survival rates at 100 days after MI were 67% for control mice, 22% for mice that received Dox alone, and 56% for mice that received Dox + Exer.

**Conclusions:**

Exercise inhibited both early and late Dox‐induced cardiotoxicity and increased recovery from an ischemic event. Exercise interventions have the potential to decrease Dox‐induced cardiac morbidity.

## Graphical abstract

This study indicates that exercise prevents Doxorubicin‐induced vascular damage, maintaining cardiac blood flow. Exercise initiated during or after Doxorubicin therapy inhibited the acute and late cardiotoxicities and increased recovery from an ischemic event.

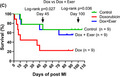

## 1 INTRODUCTION

Doxorubicin (Dox) is an effective chemotherapy for treating sarcoma in children, adolescents, and young adults (AYAs).^1^ However, Dox is associated with dose‐dependent cardiotoxicity.^2^, ^3^ Approximately 57% of survivors experience late‐onset cardiotoxicity and heart failure,^4^ which compromises quality of life (QOL) and longevity. Various approaches to limit Dox‐induced cardiac damage without compromising treatment efficacy have been investigated, but shown only minimal impact.

Exercise prevents cardiotoxicity and improves cardiac risk factors in breast cancer survivors.^5^, ^6^ Exercise has been shown to be feasible for pediatric cancer patients during treatment.^7^, ^8^, ^9^, ^10^ We hypothesized that exercise interventions initiated during and after Dox therapy will decrease acute cardiotoxicity, resulting in a reduction in late cardiac morbidity and improved long‐term survival. Previous studies done in mice and rats have not been consistent in terms of showing Dox‐induced cardiac toxicity.^11^, ^12^, ^13^, ^14^ However, these models used “adult‐equivalent” mice. Because the developing juvenile heart is more susceptible to Dox‐induced damage, and anthracycline‐induced cardiotoxicity is 3 times more frequent in females,^15^ we developed a juvenile cardiotoxicity mouse model using 4‐week‐old female mice. We showed that exercise during Dox therapy protected against *acute* Dox‐induced cardiotoxicity immediately after therapy, without decreasing tumoricidal efficacy.^16^, ^17^

We extended these investigations to determine whether exercise during Dox therapy also decreases *late*‐*onset* cardiotoxicity, using echocardiography to evaluate functional changes in ejection fraction (EF), FS, and systolic and diastolic functions. We also evaluated histologic changes in the heart tissue which are associated with cardiac damage (autophagy, abnormal mitochondria, fibrosis, and abnormal cardiac vascular structure). In addition, we investigated the effect of initiating exercise *after* Dox therapy to determine whether this intervention has the potential to stimulate tissue repair and thereby decrease late cardiotoxicity, resulting in an increased ability to recover from an ischemic event. This would be a beneficial strategy for bone sarcoma patients who may have difficulty exercising preoperatively. Our study shows that exercise done *during* Dox therapy inhibited both *early* and *late* cardiotoxicity and improved survival following myocardial infarction (MI). Exercise initiated *after* Dox therapy also decreased late cardiotoxicity. These studies indicate that exercise interventions either during or after Dox therapy can decrease the risk of cardiac disease in childhood cancer survivors.

## 2 MATERIALS AND METHODS

**FIGURE 1:**
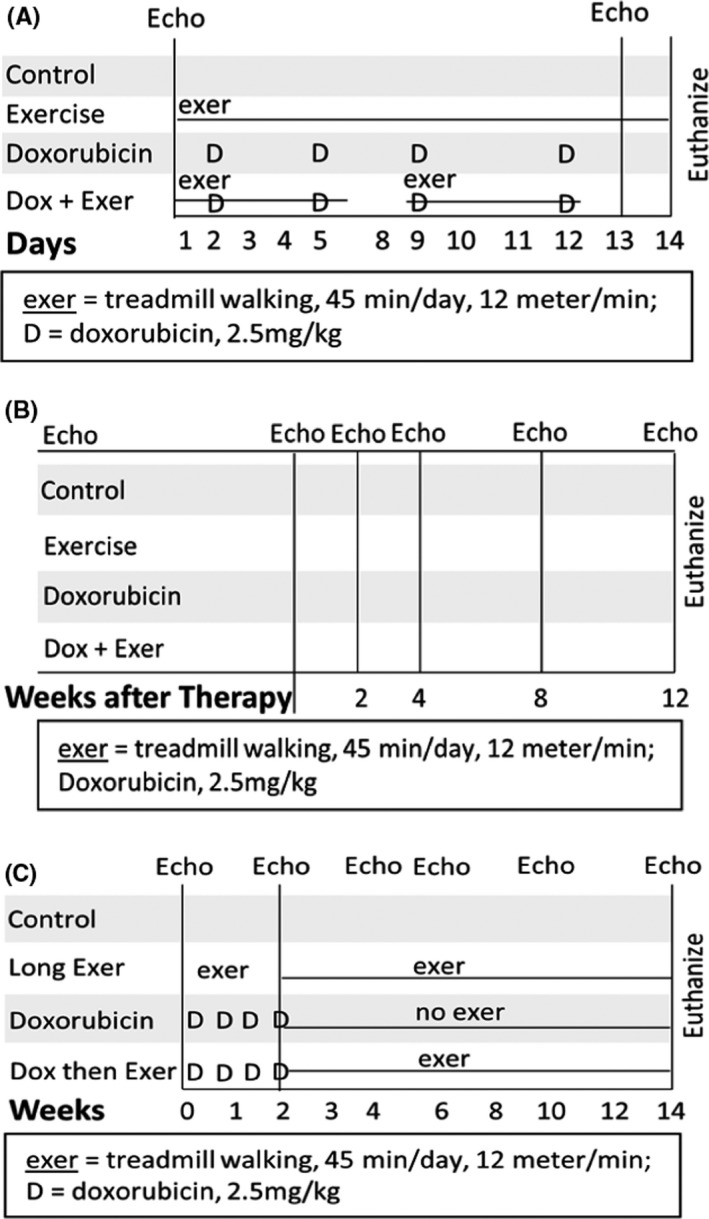
Doxorubicin (Dox)‐induced juvenile mouse cardiotoxicity model. (A) Schematic representation of the study design to assess acute Dox‐induced cardiotoxicity using nude or Balb/c mice (*n* = 8 in each group). Four‐week‐old mice were randomly divided into four groups: control, exercise alone, Dox alone, or Dox + Exer. All mice were treated by tail vein with phosphate‐buffered saline or Dox twice per week for 2 weeks. The exercise regimen was 45 min treadmill walking/day, 5 days/week. Echocardiography was conducted before treatment and 24 h after the final dose of Dox. Mice were killed 48 h after the final dose of Dox. (B) To assess Dox‐induced late cardiotoxicity, mice were treated for 2 weeks as in A and then followed them for 12 weeks after therapy. Echocardiography evaluation was done before and 24 h after therapy and then at 2, 4, 8, and 12 weeks following therapy (*n* = 10 in each group). (C) To assess the effect of initiating exercise *after* Dox therapy, mice were treated with or without Dox. The exercise protocol described in A was initiated 24 h after therapy. Echocardiography evaluation was done before and 24 h after therapy and then at 2, 4, 8, and 12 weeks after therapy (*n* = 10 in each group). Dox + Exer indicates doxorubicin + exercise

Treatment schemas are shown in Figure 1A–C and Figure S1. The methods and the associated references are provided in the Supplemental Section online.

### 2.1 Animals

Four‐week‐old *T*‐*cell*‐*deficient nude* and *immunocompetent Balb*/*c* mice were used. Cardiac functional activity was assessed by quantifying EF, FS, and left ventricular posterior wall (LVPW) thickness in the diastole and systole using echocardiography. Cardiac tissues were assessed using transmission electron microscopy (TEM), immunofluorescence, and histologic staining.

### 2.2 Statistical analyses

For any multiple‐group comparisons, one‐way ANOVA followed by Bonferroni test was performed. Two‐factor repeated‐measures ANOVA was used for mouse weights and Masson trichrome staining. Survival rates were analyzed by the Kaplan–Meier method with the log‐rank test. All values were presented as mean ± SD; *n* refers to the sample size. A value of *p* < 0.05 was considered statistically significant.

## 3 RESULTS

### 3.1 Effect of exercise on acute Dox‐induced histologic change

**FIGURE 2:**
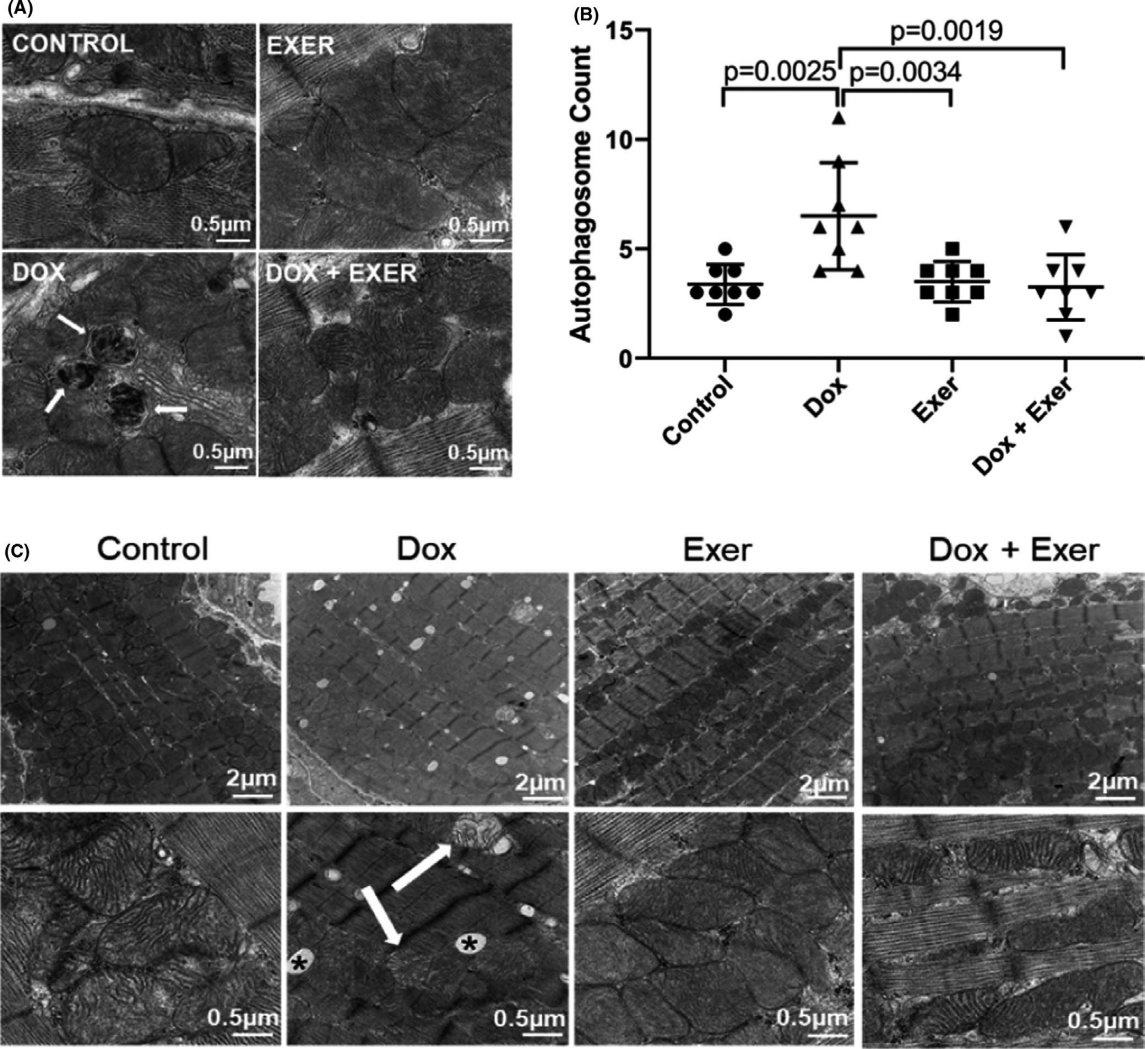
Exercise inhibited doxorubicin‐induced autophagy and abnormal mitochondria in nude mice treated with Dox for 2 weeks. (A) Representative transmission electron microscopy (TEM) images of autophagosomes (arrow) 48 h after Dox therapy. (B) Quantitative analysis of autophagosomes. (C) Representative TEM images of abnormal mitochondria (arrow) and vacuolization (asterisk). *n* = 8 mice per group. Control versus Dox, *p* = 0.0025; Dox versus Dox + Exer, *p* = 0.0034 by one‐way ANOVA followed by Bonferroni test

We previously showed that exercise during Dox treatment prevented acute Dox‐induced *functional* changes in nude and C57BL/6J immunocompetent mice.^16^ This was confirmed using Balb/c mice (Figure S2A–C). To assess whether Dox‐induced *histologic* damage was also prevented by exercise, nude and Balb/c mice were treated with Dox or Dox + Exer for 2 weeks (Figure 1A). Hearts were then harvested and evaluated by transmission electron microscopy (TEM) and immunohistochemistry (IHC). Induction of autophagy (Figure 2A), an increase in the number of autophagosomes (Figure 2B), and abnormal mitochondria (Figure 2C) (signs of cardiac damage) were observed in the hearts from mice treated with Dox but not Dox + Exer. This protective effect of exercise was also seen in Balb/c mice (Figure S3C,D).

**FIGURE 3:**
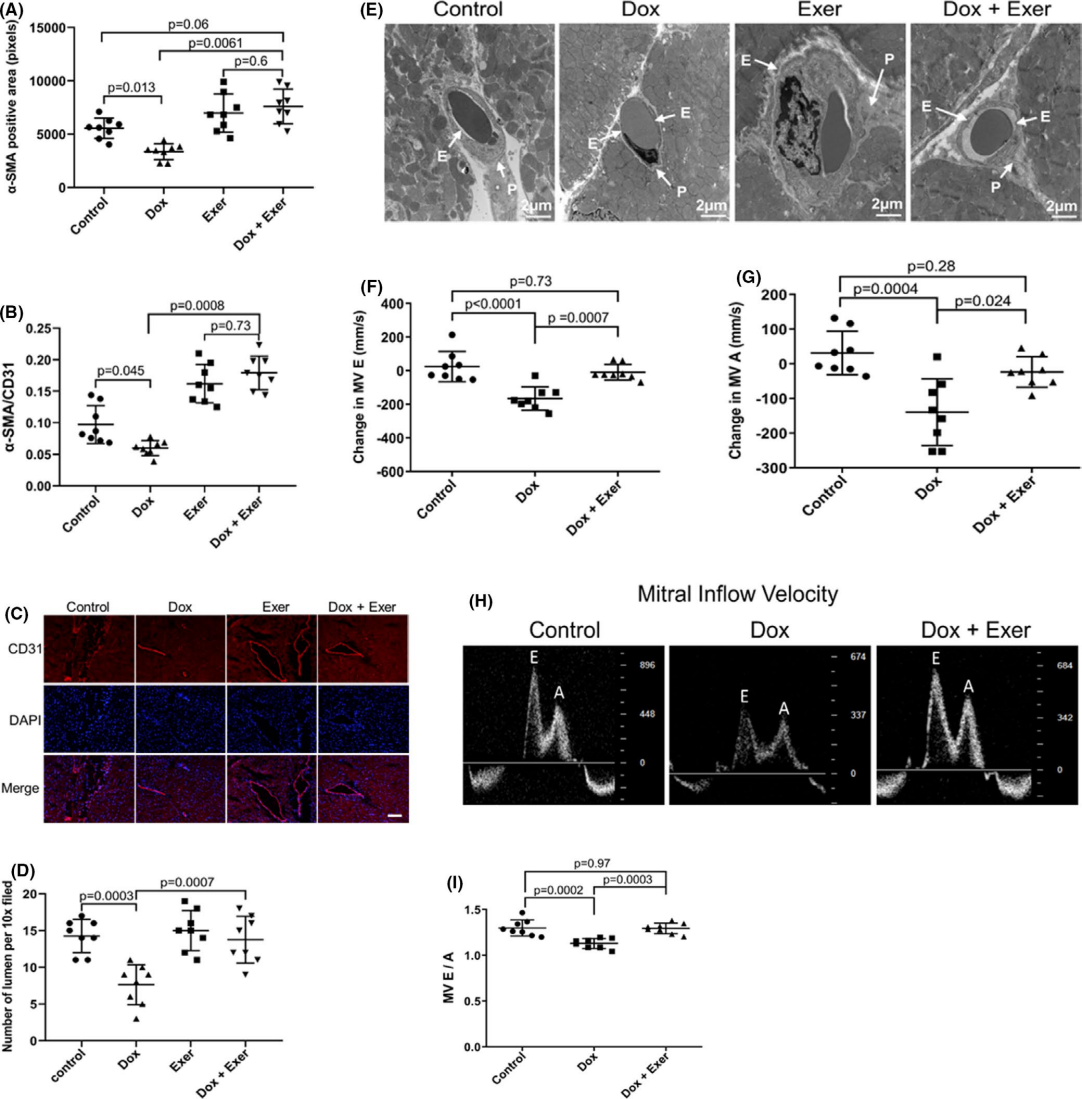
Effect of doxorubicin with and without exercise on cardiac vessel morphology and cardiac diastolic blood flow in nude mice following Dox therapy. (A) Quantitative analysis of the total area of pericytes (as defined by α‐SMA) in the heart tissues. (B) Ratio of α‐SMA/CD31 cells (mean ± SD for five random fields). (C) Representative images of CD31^+^ staining. Scale bar: 50 μm. (D) Quantitative analysis of number of open lumens in vessels >100 μm from each treatment group (mean ± SD; Control versus Dox, *p* = 0.0003; Dox versus Dox + Exer, *p* = 0.0007). Statistical analysis as described in Figure 2. (E) Representative transmission electron microscopy images of cardiac vessels. Vascular endothelial cells and pericytes are indicated by the arrow. E indicates endothelial. P indicates pericytes. (F, G) Change in mitral valve E and A velocity 2 weeks after therapy. (H) Representative echocardiography images of the E and A peak. (I) *E*/*A* ratio was measured for each mouse. Dox indicates doxorubicin and Exer indicates exercise

To investigate whether Dox affected the cardiac vessels, CD31 and α‐SMA were used to assess vessel morphology. The number of CD31^+^ vessels was not different in the Dox‐treated mice compared with controls (data not shown). However, there was a significant decrease in α‐SMA^+^ cells (Figure 3A) and the ratio of α‐SMA/CD31 (Figure 3B), a measure of vascular pericyte coverage. This suggests a deficiency in pericytes in the Dox‐treated heart vessels. There was also a significant decrease in the number of vessels >100 μm with open lumens in the Dox‐treated hearts (Figure 3C,D). The number of open lumens was significantly higher in heart tissue from mice treated with Dox + Exer compared to Dox‐treated mice (Figure 3D) and comparable to the control values.

Abnormal vascular morphology was confirmed by TEM (Figure 3E). Because pericyte coverage correlates with vessel function and efficient blood flow, these data suggest that the vessel function was compromised after Dox therapy. These vascular abnormalities were not seen in the hearts from mice treated with Dox + Exer (Figure 3A–E). Identical results were found in Balb/c mice (Figure S3A,B).

LV diastolic function was studied in the context of Dox‐induced cardiotoxicity. As the myocardium is damaged, the velocity of blood flowing into the left ventricle is reduced. We measured the velocity of left ventricular diastolic blood flow 2 weeks after therapy. Mitral valve maximum E velocity (MV E, mm/s) and mitral valve maximum A velocity (MV A, mm/s) were significantly decreased (indicating impaired blood flow) in the mice treated with Dox alone but not in mice treated with Dox + Exer (Figure 3F,G). Additional indices of left ventricular diastolic function, as assessed by mitral inflow velocity and E/A ratio, confirmed significant differences between control and Dox, but not Dox + Exer‐treated mice (Figure 3H,I).

**FIGURE 4:**
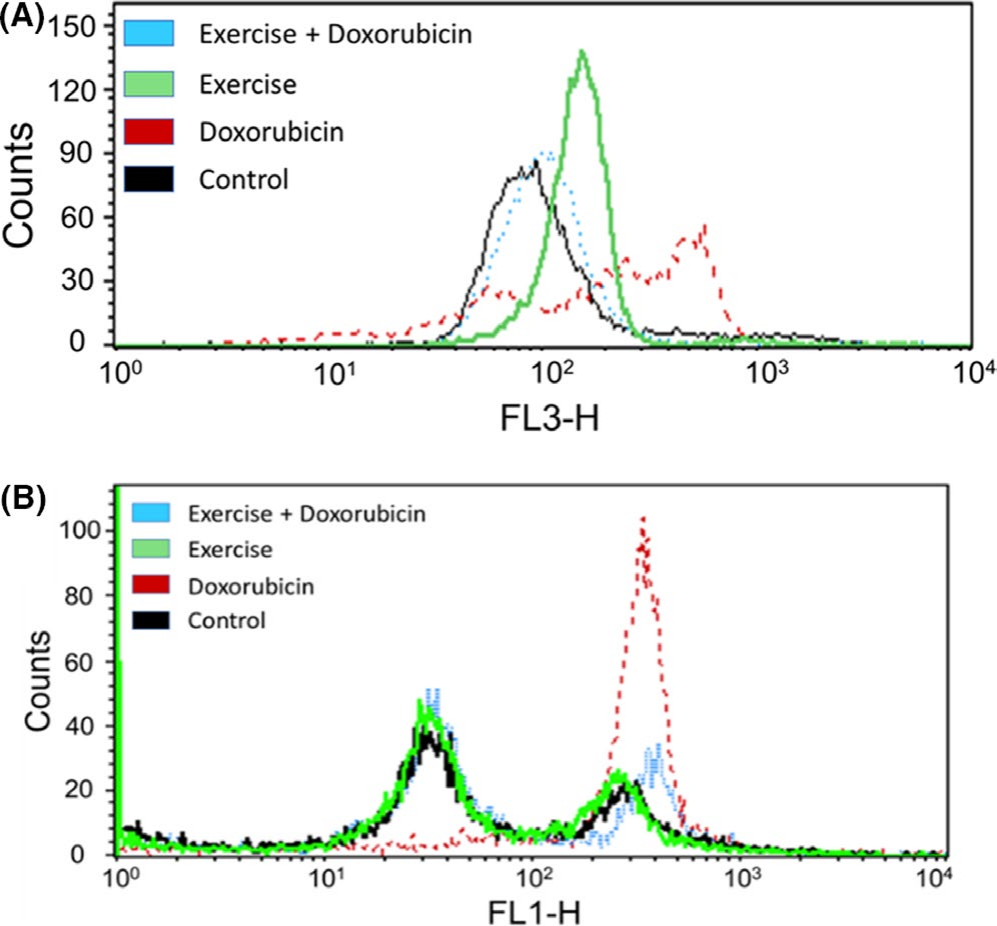
Exercise reduced doxorubicin (Dox)‐induced oxidative stress after 2 weeks in peripheral blood mononuclear cells (PBMCs) from immunodeficient and immunocompetent mice. (A) Reactive oxygen species production in PBMCs. Nude mice were treated with phosphate‐buffered saline (black line), Dox alone (red line), exercise alone (green line), or Dox + exercise (blue line). Peripheral blood was collected 24 h after therapy and PBMCs were isolated. Dihydroethidium staining was used for the measurement of intracellular superoxide using flow cytometry. Mean fluorescent intensity for dihydroethidium is shown for representative mice. (B) Balb/c mice were treated with phosphate‐buffered saline (black line), Dox alone (red line), exercise alone (green line), or Dox + exercise (blue line). Peripheral blood was collected 24 h after therapy. PBMCs were then isolated and stained for intracellular peroxides by flow cytometry using dichlorofluorescein. Mean fluorescent intensity is shown for representative mice

We also measured intracellular ROS in PBMCs. Both immunodeficient and immunocompetent mice treated with Dox had increased intracellular superoxide or peroxides (Figure 4A,B). This increase was not seen in the Dox + Exer group. These data indicate a systemic reduction of ROS by exercise.

### 3.2 Effect of exercise during therapy on late Dox‐induced cardiotoxicity

**FIGURE 5:**
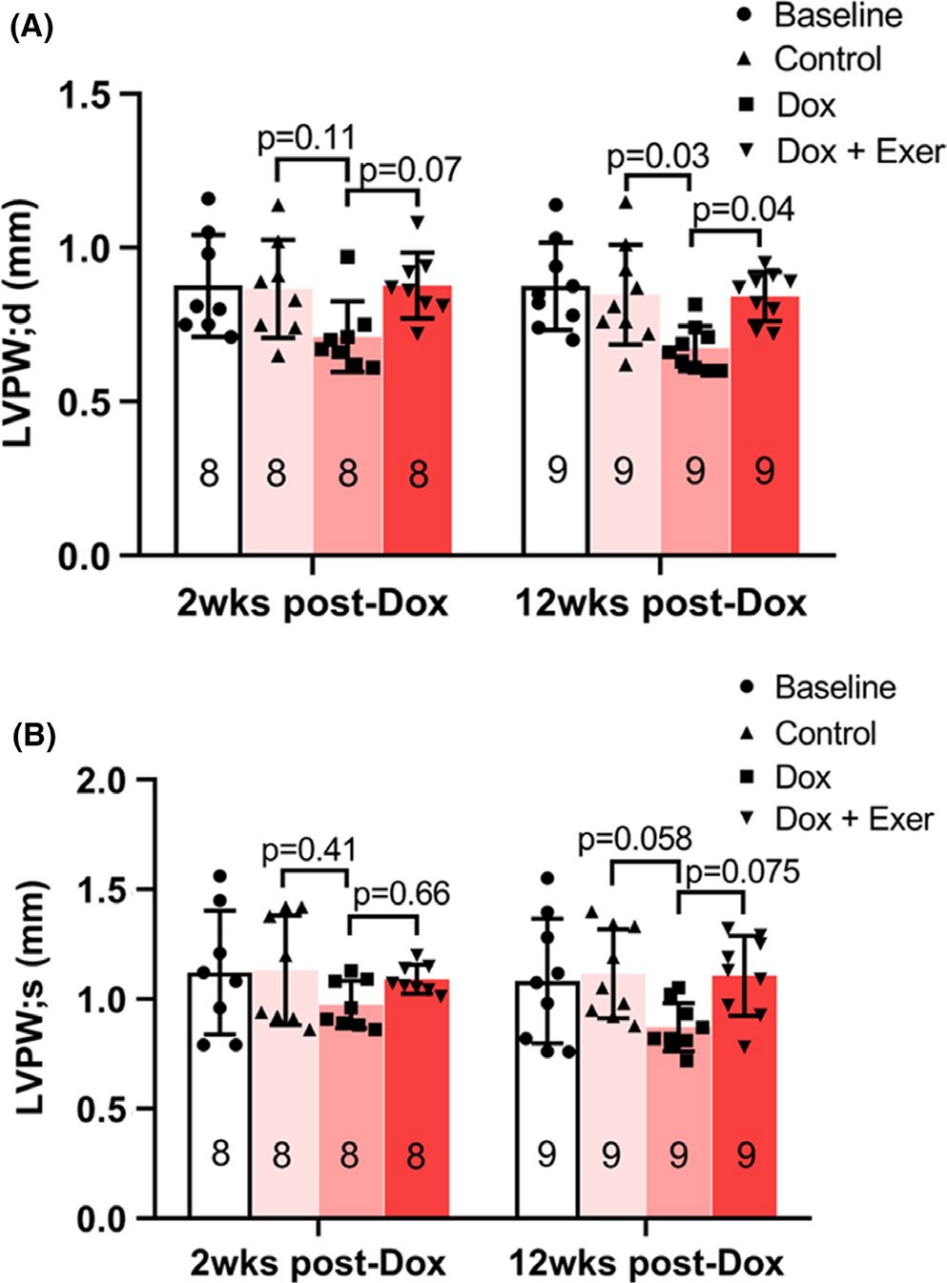
Effect of doxorubicin with or without exercise on diastolic and systolic function in nude mice 12 weeks after therapy. Diastolic (A) and systolic (B) functions were assessed after therapy and 12 weeks later using echocardiography by measuring left ventricular posterior wall thickness in diastole (LVPW; d) and systole (LVPW; s). At 2 weeks post‐Dox (*n* = 8 mice per group), control versus Dox, *p* = 0.11; Dox versus Dox + Exer, *p* = 0.07; at 12 weeks post‐Dox (*n* = 9 mice per group), control versus Dox, *p* = 0.03; Dox versus Dox + Exer, *p* = 0.04. Statistical analysis as described in Figure 2

To determine whether exercise during therapy also prevents late cardiotoxicity, diastolic and systolic functions, as quantified by LVPW thickness in diastole (d) or systole (s), were assessed after therapy and then again 12 weeks later. Although there was no significant change in LVPW thickness in either diastole or systole in the Dox‐treated group *immediately* after Dox therapy (Figure 5A,B), there was a significant decrease in LVPW thickness in *diastole* at 12 weeks (Figure 5A), indicative of late cardiotoxicity. By contrast, no change in the LVPW thickness in *diastole* was seen in the Dox + Exer group at 12 weeks. No change in LVPW thickness in systole was seen at 12 weeks in either the Dox or Dox + Exer mice (Figure 5B).

**FIGURE 6:**
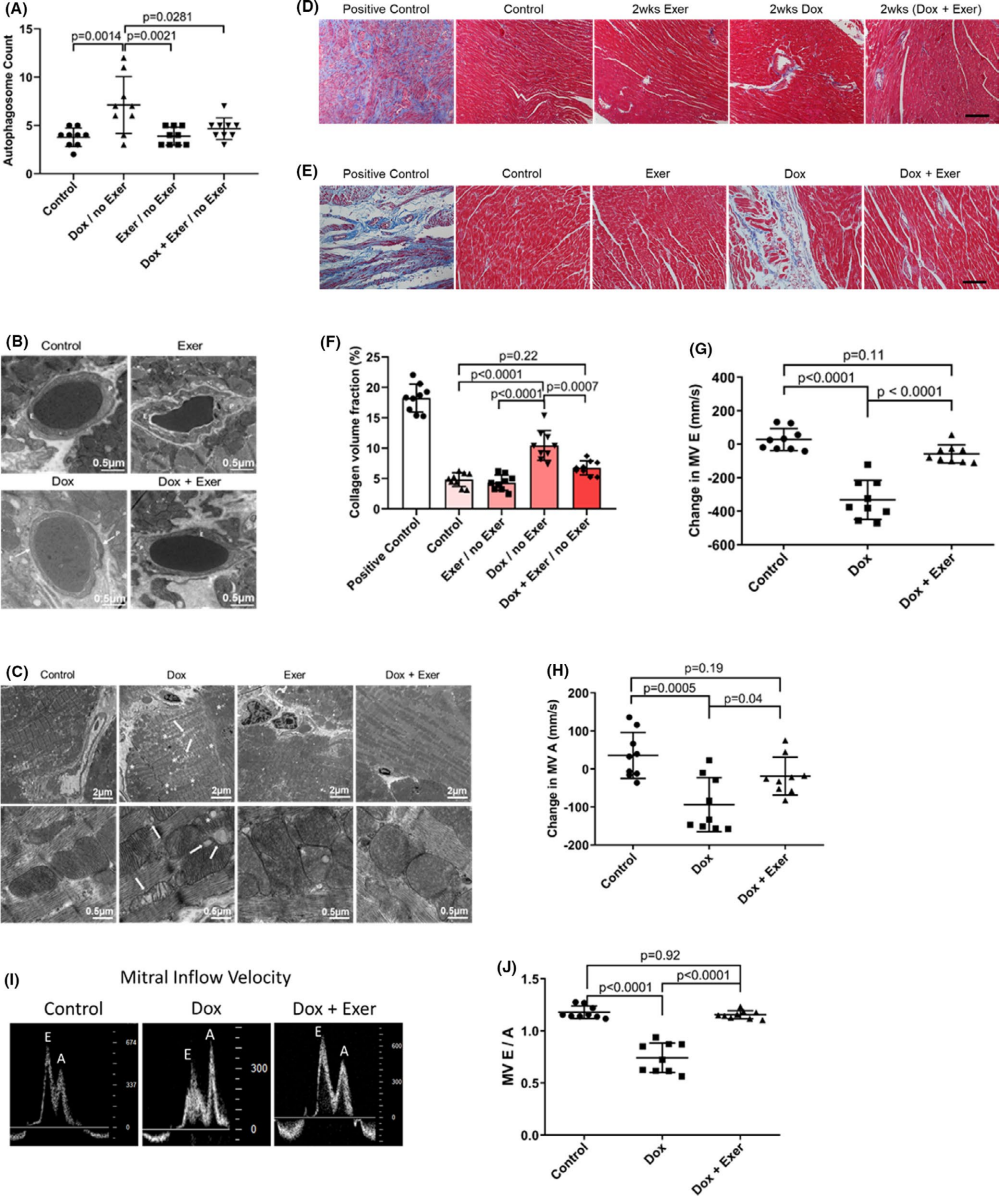
Exercise during doxorubicin (Dox) therapy prevented late Dox‐induced cardiotoxicity in nude mice 12 weeks after therapy. (A) Quantitative analysis of autophagosomes in the heart tissue. Twelve weeks after therapy, control versus Dox, *p* = 0.0014; Dox versus Dox + Exer, *p* = 0.0281. Statistical analysis as described in Figure 2. (B) Representative transmission electron microscopy (TEM) images of cardiac vessels. P indicates pericytes and E, endothelial cells. (C) Representative TEM images of abnormal mitochondria (arrow) and vacuolization (asterisk). (D, E) Collagen deposition in the left ventricle was determined using Masson trichrome staining after therapy completion (D) and 12 weeks after therapy (E). wks indicates weeks. Scale bar: 100 μm. (F) Quantification of collagen deposition by percentage collagen volume fraction (CVF%). Control versus Dox, ****p < 0.0001; Dox versus Dox + Exer, *p* = 0.0007 by two‐factor repeated‐measures ANOVA. (G, H) Change in mitral valve E and A velocity 12 weeks after therapy. (I) Representative echocardiography images of the E and A peak. (J) The E/A ratio was measured for each mouse (*n* = 9 in each group)

Cardiac histology was also analyzed 12 weeks after therapy. The increased autophagy, abnormal vessel morphology, and mitochondria (Figure 6A–C) were still evident 12 weeks after therapy in the Dox‐treated but not the Dox + Exer‐treated group. We also analyzed for cardiac fibrosis using collagen staining. Although no significant cardiac fibrosis was seen in the Dox‐treated mice *immediately* after therapy, significant collagen deposition was seen *12 weeks after therapy* (Figure 6D–F). By contrast, there was no significant fibrosis in the hearts from mice treated with Dox + Exer immediately after therapy or 12 weeks later. These data indicate that exercise during Dox therapy prevents both *acute* and *late* cardiotoxicities.

Finally, we measured the velocity of left ventricular blood flow 12 weeks after therapy. MV E and MV A velocities were significantly decreased in the Dox‐ but not the Dox + Exer‐treated mice (Figure 6G,H). Indices of left ventricular diastolic function at 12 weeks after therapy, as quantified by mitral inflow velocity and MV E/A ratio, also revealed significant differences between control versus Dox but not Dox + Exer‐treated mice (Figure 6I,J). In addition, in the Dox‐treated mice, the left ventricular diastolic function measurements 12 weeks after therapy had significantly worsened compared to the measurements taken 2 weeks after therapy (Figure 3H,I) with the E/A ratio now <1, suggesting continued deterioration of left ventricular compliance.

### 3.3 Effect of exercise after therapy on late cardiotoxicity

**FIGURE 7:**
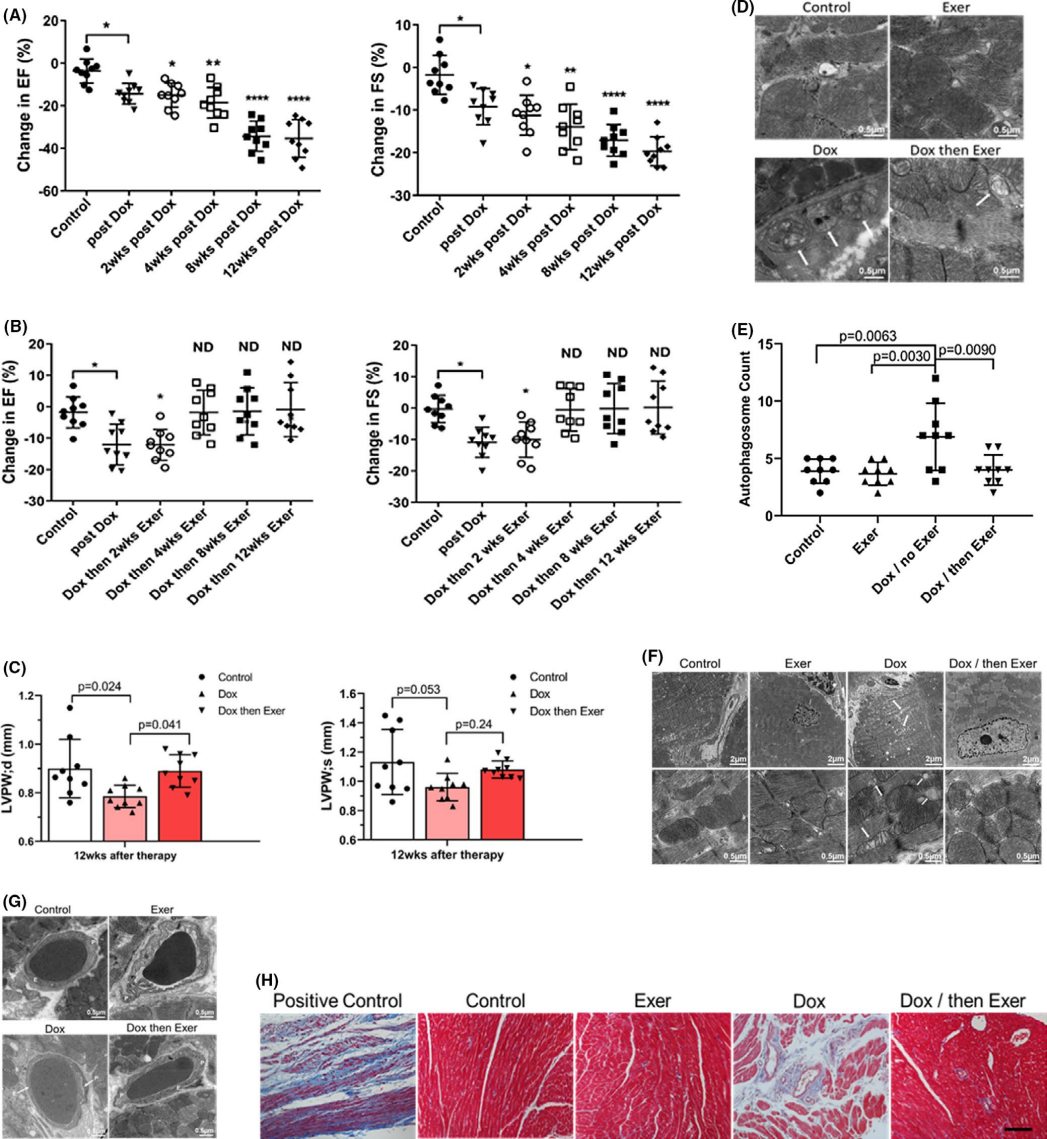
Exercise after doxorubicin (Dox) therapy inhibited long‐term Dox‐induced cardiotoxicity in nude mice. The data in Figure 7 were analyzed from the same experiment as that in Figure 6. There were six total groups in the experiment (Figure S1). The data were then presented to focus on the effect of exercise during therapy versus exercise initiated after therapy. The six individual groups in Figure S1 are Contro–l, Dox for 2 weeks and then no therapy; exercise for 2 weeks and then no therapy; and Dox + Ex for 2 weeks and. then no therapy in Figure 6. In Figure 7, the groups were from the same experiment but show the Control, Dox for 2 weeks and then no therapy; exercise for 14 weeks; and Dox for 2 weeks and then Exercise for 12 weeks. (A) Ejection fraction (EF) and fractional shortening (FS) were quantified by echocardiography 24 h after Dox therapy and then 2, 4, 8, and 12 weeks later. *p < 0.05. **p < 0.01. ****p < 0.0001. (B) EF and FS were quantified as in A in mice that exercised after Dox therapy. ND indicates no difference compared to control. *p < 0.05. (C) Left ventricular posterior wall thickness was measured in the diastole (LVPW, d) and systole (LVPW, s). In LVPW, d, control versus Dox, *p* = 0.024; Dox versus Dox and then Exer, *p* = 0.041. In LVPW, s, control versus Dox, *p* = 0.053; Dox versus Dox and then Exer, *p* = 0.24. Statistical analysis as described in Figure 2. (D) Representative transmission electron microscopy (TEM) images of autophagy (arrows). (E) Quantitative analysis of autophagosomes in the heart. (F) Representative TEM images of abnormal mitochondria (arrow) and vacuolization (asterisk) (the data from the six groups are shown altogether in Figure S4). (G) Representative TEM images of cardiac vessels (the data from the six groups are shown altogether in Figure S5). P indicates pericytes and E, endothelial cells. (H) Collagen deposition in the left ventricle was determined using Masson trichrome staining in mice that exercised after Dox therapy. Scale bar: 100 μm (*n* = 9 in each group)

Sarcoma patients may not be able to exercise prior to surgery. Therefore, we also evaluated the effectiveness of initiating exercise *after* Dox therapy in reducing late‐onset cardiotoxicity. Mice treated with Dox alone had a significant decrease in EF and FS immediately after therapy, which declined further over 8–12 weeks (Figure 7A).

By contrast, in the mice that exercised after Dox therapy, there was an acute decrease in EF and FS after Dox therapy, with no change after 2 weeks of post‐therapy exercise, but an improvement and restoration to normal values in both the EF and FS following 4–12 weeks of exercise (Figure 7B). LVPW thickness in diastole at 12 weeks was also normal (Figure 7C). The increased autophagy, abnormal mitochondria, and vascular morphology and fibrosis were seen in the Dox‐treated mice 12 weeks after therapy but not in the mice that exercised after Dox (Figure 7D–H, Figures S4 and S5).

### 3.4 Recovery following MI

**FIGURE 8:**
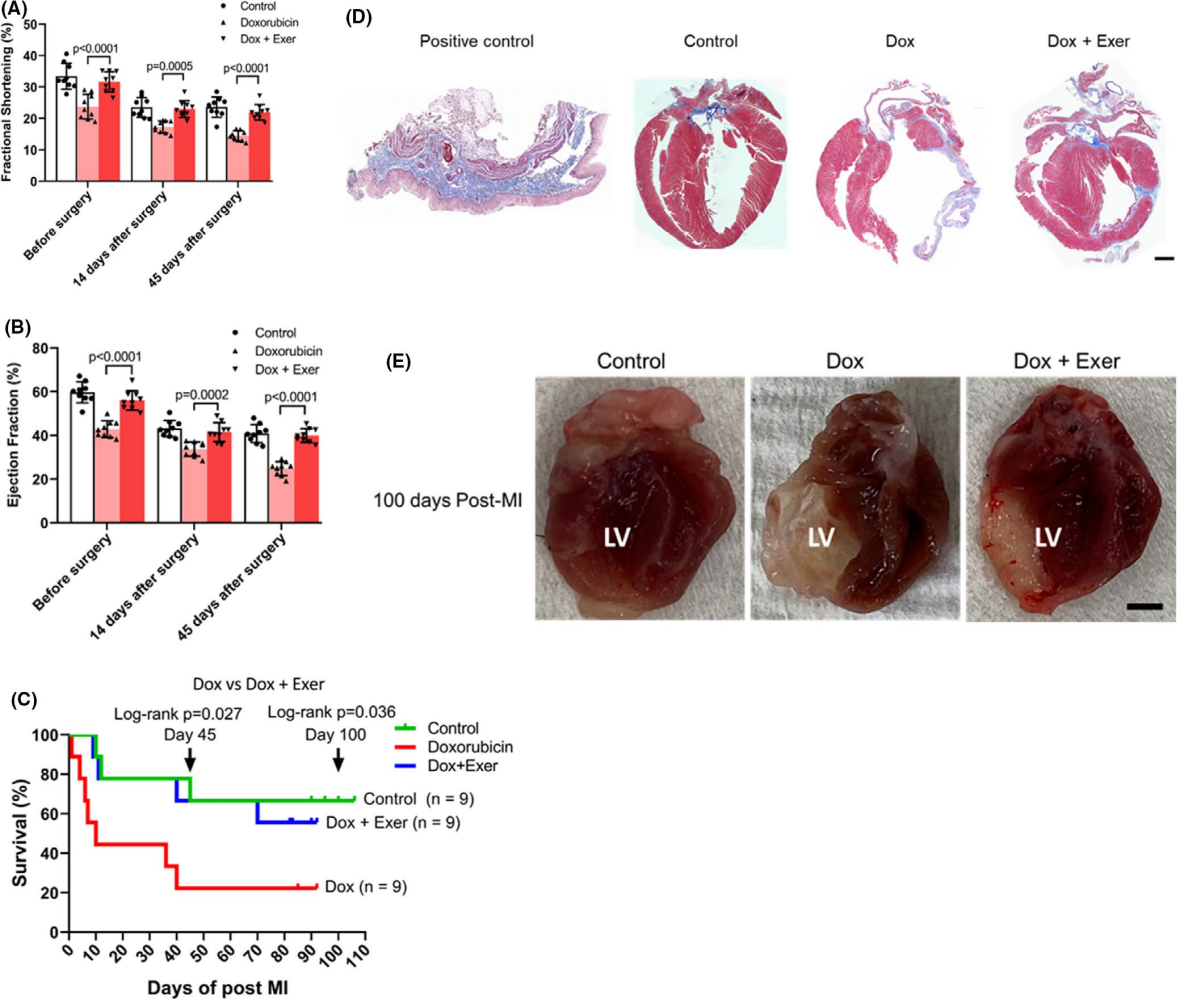
Recovery following left anterior descending artery ligation (myocardial infarction). Myocardial infarction (MI) surgery was performed 90 days after treatment with doxorubicin (Dox), Dox + exercise (Exer), or no therapy (control). (A, B) Fractional shortening (FS) and ejection fraction (EF) were assessed before surgery and then 14 and 45 days after surgery. ****p < 0.0001. Statistical analysis as described in Figure 2. (C) Survival rates were quantified following MI for each group. Kaplan–Meier survival curves of mice at day 45 and day 100 (arrows) after MI. *n* indicates number of mice. *p < 0.05. (D) Collagen deposition in the left ventricle was assessed using Masson trichrome staining 100 days after MI. Mice treated with Dox alone had a thinned left ventricle with collagen deposition in the left ventricular wall. This was not observed in the mice treated with Dox + Exer. Scale bar: 0.5 mm. (*n* = 9 in each group). (E) Left ventricular wall heart tissue following myocardial infarction (MI) for 100 days in nude mice. LV indicates left ventricle. Dox indicates doxorubicin. Exer indicates exercise

Acute cardiac damage can compromise the ability of the heart to recover from a cardiac insult. Dox exposure can therefore sensitize the heart to cardiac challenges, impeding its ability to recover from an insult. To assess whether an exercise intervention would improve recovery from a cardiac insult, we performed ligation of the LAD artery to mimic an MI 90 days after Dox alone or Dox + Exer therapy. Control mice had no therapy prior to MI. Although FS and EF decreased in all three groups 14 days after MI, the decrease in FS and EF in Dox‐treated mice was significantly greater than both the control and Dox + Exer groups at 14 and 45 days (Figure 8A,B).

Survival was monitored for 100 days post‐MI. Dox therapy significantly reduced survival compared to control untreated mice subjected to MI (22% compared with 67%; *p* < 0.05). The survival rate of Dox + Exer‐treated mice was significantly improved compared to Dox‐treated mice at both 45 days (67% compared with 22%; *p* < 0.05) and 100 days (56% compared with 22%; *p* < 0.05; Figure 8C).

Survival of Dox + Exer‐treated mice was not significantly different from that of the control group (Figure 8C; *p* = 0.88 at day 45; *p* = 0.82 at day 100). Thinned and collapsed left ventricular walls with significant fibrosis were seen 100 days post‐MI in the Dox group but not in the control or Dox + Exer groups (Figure 8D,E).

In the echocardiogram views, Dox therapy significantly decreased left ventricular contractility, relaxation, compliance, and cardiac reserve function. Left ventricular wall heart tissue following MI showed that the left ventricle was enlarged, the left ventricular internal thickness was increased, and the LVPW and left ventricular anterior wall were significantly thinner than hearts from mice in the control or Dox + Exer groups (Video S1A–C).

## 4 DISCUSSION

Dox‐induced cardiotoxicity can be detected during therapy, within the first year following therapy, or have late‐onset.^18^ Using our juvenile cardiotoxicity model, we previously reported that an exercise intervention in both tumor‐bearing and nontumor‐bearing mice initiated during Dox therapy prevented the decrease in EF and FS seen immediately after Dox therapy.^16^ We expanded our investigations to determine whether exercise during Dox therapy also prevented the structural changes and histologic evidence of *acute* cardiac damage and reduced *late*‐*onset* cardiotoxicity. The present study was performed using *both* immunodeficient and immunocompetent mice. Results were the same. Exercise inhibited the Dox‐induced functional and structural changes indicative of cardiotoxicity in nude (Figures 2, 3, 4, 5, 6, 7, 8) and Balb/c mice (Figures S2 and S3A–E). Two weeks of Dox therapy resulted in decreased cardiac diastolic blood flow (Figure 3F,G) and the induction of autophagy and abnormal mitochondria in the heart tissue (Figure 2). The cardiac vascular morphology showed punctate vessels with decreased pericytes (Figure 3A–E). Hearts from mice that exercised during Dox therapy showed none of these abnormalities, indicating that exercise during Dox therapy prevented acute Dox‐induced cardiac damage.

Similar to Huang et al.,^2^ we found that Dox therapy had late effects on cardiac function 12 weeks after therapy. Mice treated with Dox alone showed deterioration in EF and FS over the 12‐week observation period, whereas mice treated with Dox + Exer maintained normal cardiac function with no decrease in EF or FS. No change in diastolic or systolic function or evidence of cardiac fibrosis was seen immediately after Dox therapy. However, decreased diastolic function (as determined by MV E and A waves in diastole), with slow left ventricular relaxation producing an E/A ratio <1, was seen 12 weeks after therapy in the Dox group. Cardiac tissues at this time showed development of fibrosis, continued elevation of autophagosomes, and decreased pericytes in the cardiac vasculature. Cardiac blood flow was also compromised. By contrast, in the Dox + Exer group, there was no development of early *or* late fibrosis, and normal blood flow was observed, along with no change in diastolic function. In addition, the number of autophagosomes and cardiac vessel morphology were also indistinguishable from the hearts from control mice. These functional and anatomic findings indicate that combining an exercise intervention with Dox therapy has the potential to not only decrease *acute* Dox‐induced cardiotoxicity but also prevent *late* cardiomyopathy that compromises the cardiac function of childhood cancer survivors.

We observed increased autophagosomes immediately after therapy. Autophagy is a mechanism of cell preservation following injury. Induction of autophagy in the myocardium leading to an increase in autophagosomes is a response to Dox therapy.^19^, ^20^ Thus, we expected to see the induction of autophagy immediately after therapy. However, our findings showing the persistence of elevated numbers of autophagosomes in the myocardium 12 weeks after therapy was unexpected. It is unclear whether the continued presence of increased numbers of autophagosomes was due to continued induction of autophagy in the weeks after therapy or to a block in lysosomal acidification and function, which then leads to a decrease in autophagic flux, as was shown by Li et al.^21^ Dox has been shown to block cardiomyocyte autophagic flux by inhibiting lysosome acidification leading to the accumulation of autophagosomes. The enhanced initiation of autophagy increases Dox cardiotoxicity.^21^ These authors demonstrated that inhibiting the initiation of autophagy using Beclin1+/‐ mice (that have diminished autophagic activity) protected against Dox‐induced cardiotoxicity. We demonstrated that Dox treatment increased autophagy and the number of autophagosomes in the heart. However, this was not seen in the hearts from mice treated with Dox + Exercise. We used autophagy as an indication of Dox‐induced cardiac damage. The Dox‐induced increase in autophagosomes was not seen when mice exercised during Dox. Our data are consistent with the findings of Li et al. in which inhibiting autophagy decreased Dox‐induced cardiotoxicity.

Exercise has been demonstrated to induce autophagy.^22^ However, we did not see evidence of increased autophagy in the exercise alone treated mice. Our model was different as mice only exercised for 2 weeks. This may not be equivalent to endurance exercised or exercise training and thus may explain why we did not see induction of autophagy in the group of mice that exercised alone. Under normal conditions, autophagy helps cells survive by clearing and reusing intracellular components. In contrast, if persistent stress induces an excessive degree of autophagy (as in the case of Dox therapy), autophagy may induce necrosis and apoptosis and this increased autophagy is responsible for cell death.^21^ Exercise during Dox therapy prevented both the *early* and *late* increases in autophagosomes, consistent with our findings of conserved cardiac function.

We also demonstrated that exercise initiated *after* Dox therapy has the potential to induce cardiac repair. In these experiments, mice were treated with Dox for 2 weeks, and then an exercise intervention was initiated *after* therapy completion in one group. The control group did not exercise after Dox (sedentary). Echocardiography was used to monitor both groups. As anticipated, there was a decrease in both EF and FS at the end of Dox therapy in both groups. In the Dox/sedentary group, EF and FS continued to decline over the 12‐week observation period. Although there was a similar depression in EF and FS in the “exercise intervention group” at the end of Dox therapy prior to the initiation of the exercise intervention (documenting acute Dox‐induced cardiotoxicity), exercise appeared to induce functional improvement and cardiac repair, because both EF and FS started improving after 6 weeks of exercise. EF and FS were back to pretherapy values after 8 weeks of exercise. In the mice treated with Dox alone, the LVPW thickness in the diastole was significantly decreased 12 weeks after therapy, suggesting early signs of diastolic dysfunction. However, this was not seen in the Dox/then exercise group. The LVPW thickness in the diastole in this group was not significantly different from that of the control group. We found no changes in systolic function at 12 weeks in either the Dox/sedentary or Dox/exercise groups. There was a suggestion that the systolic function was decreased in the Dox/sedentary group, but this was not statistically significant. Re‐evaluation of the systolic function beyond 14 weeks may reveal significant differences.

In addition to having normal cardiac function in the Dox/exercise mice, there was no persistence of Dox‐induced autophagy. Cardiac vessels in these mice were similar to those of control mice, and there was minimal fibrosis at 12 weeks. These results indicate that there was anatomic restoration in the hearts of mice that exercised after therapy, in addition to recovery of ventricular performance. Therefore, initiating exercise after structural changes have been detected may halt progression of cardiac remodeling and improve function. Although we documented *functional damage* in both groups *immediately* after Dox therapy, both EF and FS improved over time in the Dox/exercise mice, suggesting that an exercise intervention initiated *after* Dox therapy has the potential to decrease the intensity of late cardiomyopathies.

To confirm that exercise reduces sensitivity to physiological and pathological insults, we determined the survival rate of control, Dox alone, and Dox + Exer treated mice following ligation of the LAD artery (MI). Dox therapy significantly reduced survival following MI compared with the control and Dox + Exer‐treated mice. Survival rates of mice treated with Dox + Exer were not significantly different from those of control mice, confirming our conclusion that exercise protected the heart from both *acute* and *late* Dox‐induced cardiotoxicities.

The mechanism(s) responsible for the reduced cardiotoxicity in the mice that exercised is not a focus of this study, but we did show that levels of ROS in PBMCs from mice treated with Dox alone were significantly elevated. By contrast, levels of ROS in mice that exercised during Dox therapy were similar to those in control, untreated mice. The generation and accumulation of ROS has been implicated in the pathogenesis of Dox cardiotoxicity.^23^, ^24^, ^25^ Because of poor myocardial concentrations of superoxide dismutase, catalase, and glutathione peroxidase, these free radicals cause extensive lipid peroxidation and mitochondrial destruction. Therefore, the cardioprotective effects of exercise may be linked to its ability to reduce Dox‐induced ROS generation in the heart. In addition, induction of autophagy in the heart has also been shown to increase ROS production.^26^ We demonstrated that exercise decreased autophagy, and this may have contributed to the decrease in ROS production in the mice that exercised during therapy.

Our model was designed to look at cardiotoxicity in *juvenile* mice and the efficacy of an exercise intervention. The relevance of our mouse model to what occurs in patients is supported by our recent retrospective analysis of cardiac function in 18 AYAs patients with osteosarcoma.^27^ These patients received >250 mg/m^2^ Dox. Two years after diagnosis, there was a significant decrease in EF, E‐wave velocity, E/A ratio, and LVPW thickness end diastolic dimension, indicative of early compromised diastolic function. The echocardiogram markers of Dox‐induced cardiotoxicity and diastolic dysfunction seen in our mouse model were similar to those found in our retrospective study of AYAs patients with sarcoma.^27^ This confirms the relevance of our mouse model in defining early echocardiogram and biomarkers that can predict Dox‐induced cardiac damage and investigating the mechanisms involved in *acute* and *late* Dox‐induced cardiotoxicity.

At the present time, clinical trials aimed at preventing Dox‐induced ventricular remodeling and late cardiotoxicity in childhood and AYA cancer survivors involve the use of agents that have shown success in treating adults and children with *nononcologic*‐related heart failure.^28^, ^29^, ^30^ There are no preclinical models that confirm or refute the activity of ACE‐inhibitors, β‐blockers, or agents that block α1, β1, and β2 adrenergic activation for prevention or reversal of *Dox*‐*induced* cardiotoxicity and cardiac remodeling leading to ventricular failure. Although many of these agents have been shown to be safe in children,^31^, ^32^ the mechanisms responsible for Dox‐induced heart damage may not be identical to those leading to cardiac failure in children with Duchenne muscular dystrophy, those undergoing heart transplantation, or those with heart failure not associated with cancer and chemotherapy administration. It is imperative to use relevant preclinical models in mice of the appropriate age and sex to evaluate prospective interventions to be used with or after Dox therapy. It is equally imperative to use preclinical data to justify clinical trials aimed at decreasing *early* and *late* Dox‐induced cardiotoxicity. Such findings can help in the optimal design of a randomized trial to assess efficacy, rather than arbitrarily choosing the dose and frequency of the intervention. Including a prolonged latency period after therapy completion is the most relevant for ascertaining efficacy for survivors.

Exercise is a cost‐effective intervention that can be administered remotely, making it highly attractive for use in children and AYAs with cancer. Our preclinical studies support including an exercise intervention as part of the therapeutic approach for treating sarcoma in children and AYAs, particularly since our previous investigations showed that combining exercise with Dox therapy in mice with Ewing sarcoma decreased acute Dox‐induced heart damage *without* decreasing tumor efficacy or increasing Dox concentration in the heart.^16^, ^17^, ^33^ Late‐onset cardiomyopathies are associated with significant morbidity. Therefore, interventions to mitigate Dox‐induced cardiac damage can decrease mortality and improve QOL for childhood and AYA cancer survivors.

## CONFLICTS OF INTEREST

The authors declare no conflicts of interest.

## ETHICAL STATEMENT

All animal experiments were approved by University of Texas MD Anderson Cancer Center (MDACC) Institutional Animal Care and Use Committee (IACUC) that conform (approval reference No. 00001615‐RN01) to the Guide for the Care and Use of Laboratory Animals published by the United States National Institutes of Health (NIH, Eighth edition, revised 2011). Nude mice were purchased from Experimental Radiation Oncology Breeding core of MDACC. Balb/c mice were purchased from NCI with Charles River Laboratories (US).

## Supporting information

Supplementary Material

## Data Availability

The data that support the findings of this study are available from the corresponding author upon reasonable request.

## References

[1] Smith MA , Ungerleider RS , Horowitz ME , Simon R . Influence of doxorubicin dose intensity on response and outcome for patients with osteogenic sarcoma and Ewing's sarcoma. J Natl Cancer Inst. 1991;83(20):1460‐1470. 1833556 10.1093/jnci/83.20.1460

[2] Huang C , Zhang X , Ramil JM , et al. Juvenile exposure to anthracyclines impairs cardiac progenitor cell function and vascularization resulting in greater susceptibility to stress‐induced myocardial injury in adult mice. Circulation. 2010;121(5):675‐683. 20100968 10.1161/CIRCULATIONAHA.109.902221 PMC2834271

[3] Lindsey ML , Lange RA , Parsons H , Andrews T , Aune GJ . The tell‐tale heart: molecular and cellular responses to childhood anthracycline exposure. Am J Physiol Heart Circ Physiol. 2014;307(10):H1379‐H1389. 25217655 10.1152/ajpheart.00099.2014 PMC4233297

[4] Paulides M , Kremers A , Stöhr W , et al.; German Late Effects Working Group in the Society of Pediatric O, Haematology . Prospective longitudinal evaluation of doxorubicin‐induced cardiomyopathy in sarcoma patients: a report of the late effects surveillance system (LESS). Pediatr Blood Cancer. 2006;46(4):489‐495. 16333817 10.1002/pbc.20492

[5] Mishra SI , Scherer RW , Snyder C , Geigle P , Gotay C . Are exercise programs effective for improving health‐related quality of life among cancer survivors? A systematic review and meta‐analysis. Oncol Nurs Forum. 2014;41(6):E326‐E342. 25355029 10.1188/14.ONF.E326-E342 PMC4332787

[6] Kirkham AA , Davis MK . Exercise prevention of cardiovascular disease in breast cancer survivors. J Oncol. 2015;2015:917606. 26339243 10.1155/2015/917606 PMC4539168

[7] Huang TT , Ness KK . Exercise interventions in children with cancer: a review. Int J Pediatr. 2011;2011:1‐11. 10.1155/2011/461512 PMC3205744 22121378

[8] Braam KI , van der Torre P , Takken T , Veening MA , van Dulmen‐den BE , Kaspers GJ . Physical exercise training interventions for children and young adults during and after treatment for childhood cancer. Cochrane Database Syst Rev. 2016;3:CD008796. 27030386 10.1002/14651858.CD008796.pub3 PMC6464400

[9] Esbenshade AJ , Friedman DL , Smith WA , et al. Feasibility and initial effectiveness of home exercise during maintenance therapy for childhood acute lymphoblastic leukemia. Pediatr Phys Ther. 2014;26(3):301‐307. 24979081 10.1097/PEP.0000000000000053 PMC4211618

[10] Baumann FT , Bloch W , Beulertz J . Clinical exercise interventions in pediatric oncology: a systematic review. Pediatr Res. 2013;74(4):366‐374. 23857296 10.1038/pr.2013.123

[11] Hiroe M , Ohta Y , Fujita N , et al. Myocardial uptake of 111In monoclonal antimyosin Fab in detecting doxorubicin cardiotoxicity in rats. Morphological and hemodynamic findings. Circulation. 1992;86(6):1965‐1972. 1451268 10.1161/01.cir.86.6.1965

[12] Li K , Sung RYT , Huang WZ , et al. Thrombopoietin protects against in vitro and in vivo cardiotoxicity induced by doxorubicin. Circulation. 2006;113(18):2211‐2220. 16651473 10.1161/CIRCULATIONAHA.105.560250

[13] Sawyer DB , Fukazawa R , Arstall MA , Kelly RA . Daunorubicin‐induced apoptosis in rat cardiac myocytes is inhibited by dexrazoxane. Circ Res. 1999;84(3):257‐265. 10024299 10.1161/01.res.84.3.257

[14] Imondi AR , Della Torre P , Mazue G , et al. Dose‐response relationship of dexrazoxane for prevention of doxorubicin‐induced cardiotoxicity in mice, rats, and dogs. Cancer Res. 1996;56(18):4200‐4204. 8797592

[15] Lipshultz SE , Lipsitz SR , Mone SM , et al. Female sex and higher drug dose as risk factors for late cardiotoxic effects of doxorubicin therapy for childhood cancer. N Engl J Med. 1995;332(26):1738‐1743. 7760889 10.1056/NEJM199506293322602

[16] Wang F , Iskra B , Kleinerman E , et al. Aerobic exercise during early murine doxorubicin exposure mitigates cardiac toxicity. J Pediatr Hematol Oncol. 2018;40(3):208‐215. 29557918 10.1097/MPH.0000000000001112 PMC6876617

[17] Morrell MBG , Alvarez‐Florez C , Zhang A , et al. Vascular modulation through exercise improves chemotherapy efficacy in Ewing sarcoma. Pediatr Blood Cancer. 2019;66(9):e27835. 31136074 10.1002/pbc.27835 PMC6646082

[18] Broder H , Gottlieb RA , Lepor NE . Chemotherapy and cardiotoxicity. Rev Cardiovasc Med. 2008;9(2):75‐83. 18660728 PMC3723407

[19] Lu L , Wu W , Yan J , Li X , Yu H , Yu X . Adriamycin‐induced autophagic cardiomyocyte death plays a pathogenic role in a rat model of heart failure. Int J Cardiol. 2009;134(1):82‐90. 18619688 10.1016/j.ijcard.2008.01.043

[20] Kobayashi S , Volden P , Timm D , Mao K , Xu X , Liang Q . Transcription factor GATA4 inhibits doxorubicin‐induced autophagy and cardiomyocyte death. J Biol Chem. 2010;285(1):793‐804. 19901028 10.1074/jbc.M109.070037 PMC2804228

[21] Li DL , Wang ZV , Ding G , et al. Doxorubicin blocks cardiomyocyte autophagic flux by inhibiting lysosome acidification. Circulation. 2016;133(17):1668‐1687. 26984939 10.1161/CIRCULATIONAHA.115.017443 PMC4856587

[22] Lee Y , Kwon I , Jang Y , Song W , Cosio‐Lima LM , Roltsch MH . Potential signaling pathways of acute endurance exercise‐induced cardiac autophagy and mitophagy and its possible role in cardioprotection. J Physiol Sci. 2017;67(6):639‐654. 28685325 10.1007/s12576-017-0555-7 PMC5684252

[23] Singal PK , Iliskovic N . Doxorubicin‐induced cardiomyopathy. N Engl J Med. 1998;339(13):900‐905. 9744975 10.1056/NEJM199809243391307

[24] Sequeira CM , Martins MA , Alves R , et al. Aerobic exercise training attenuates doxorubicin‐induced ultrastructural changes in rat ventricular myocytes. Life Sci. 2021;264:118698. 33137370 10.1016/j.lfs.2020.118698

[25] Lee Y , Min K , Talbert EE , et al. Exercise protects cardiac mitochondria against ischemia‐reperfusion injury. Med Sci Sports Exerc. 2012;44(3):397‐405. 21857373 10.1249/MSS.0b013e318231c037

[26] Essick EE , Sam F . Oxidative stress and autophagy in cardiac disease, neurological disorders, aging and cancer. Oxid Med Cell Longev. 2010;3(3):168‐177. 20716941 10.4161/oxim.3.3.2 PMC2952075

[27] Gilchrist SC , Roth M , Livingston JA , Hildebrandt MAT , Kleinerman ES , Banchs J . Short‐term changes in cardiac function in osteosarcoma patients receiving anthracyclines. J Adolesc Young Adult Oncol. 2019;8(3):385‐386. 30794081 10.1089/jayao.2018.0141 PMC6909714

[28] Duboc D , Meune C , Pierre B , et al. Perindopril preventive treatment on mortality in Duchenne muscular dystrophy: 10 years’ follow‐up. Am Heart J. 2007;154(3):596‐602. 17719312 10.1016/j.ahj.2007.05.014

[29] Remme WJ , Riegger G , Hildebrandt P , et al. The benefits of early combination treatment of carvedilol and an ACE‐inhibitor in mild heart failure and left ventricular systolic dysfunction. The carvedilol and ACE‐inhibitor remodelling mild heart failure evaluation trial (CARMEN). Cardiovasc Drugs Ther. 2004;18(1):57‐66. 15115904 10.1023/B:CARD.0000025756.32499.6f

[30] Duboc D , Meune C , Lerebours G , Devaux JY , Vaksmann G , Becane HM . Effect of perindopril on the onset and progression of left ventricular dysfunction in Duchenne muscular dystrophy. J Am Coll Cardiol. 2005;45(6):855‐857. 15766818 10.1016/j.jacc.2004.09.078

[31] Shaddy RE , Boucek MM , Hsu DT , et al. Carvedilol for children and adolescents with heart failure: a randomized controlled trial. JAMA. 2007;298(10):1171‐1179. 17848651 10.1001/jama.298.10.1171

[32] Azeka E , Franchini Ramires JA , Valler C , Alcides Bocchi E . Delisting of infants and children from the heart transplantation waiting list after carvedilol treatment. J Am Coll Cardiol. 2002;40(11):2034‐2038. 12475466 10.1016/s0735-1097(02)02570-6

[33] Wang F , Schadler K , Chandra J , Kleinerman ES . Effect of exercise on acute and late onset Doxorubicin‐induced cardiotoxicity. Can Res. 2018;78(13). 10.1158/1538-7445.am2018-3008

