# Supplementary material for: Exercise intervention decreases acute and late doxorubicin‐induced cardiotoxicity
Source: Cancer Med. 2021 Sep 15;10(21):7572–84. doi: 10.1002/cam4.4283 (PMC8559466; doi:10.1002/cam4.4283)
Supplement: Supplementary file 1 — Supplementary Material [file CAM4-10-7572-s001.pptx]

## Slide 1
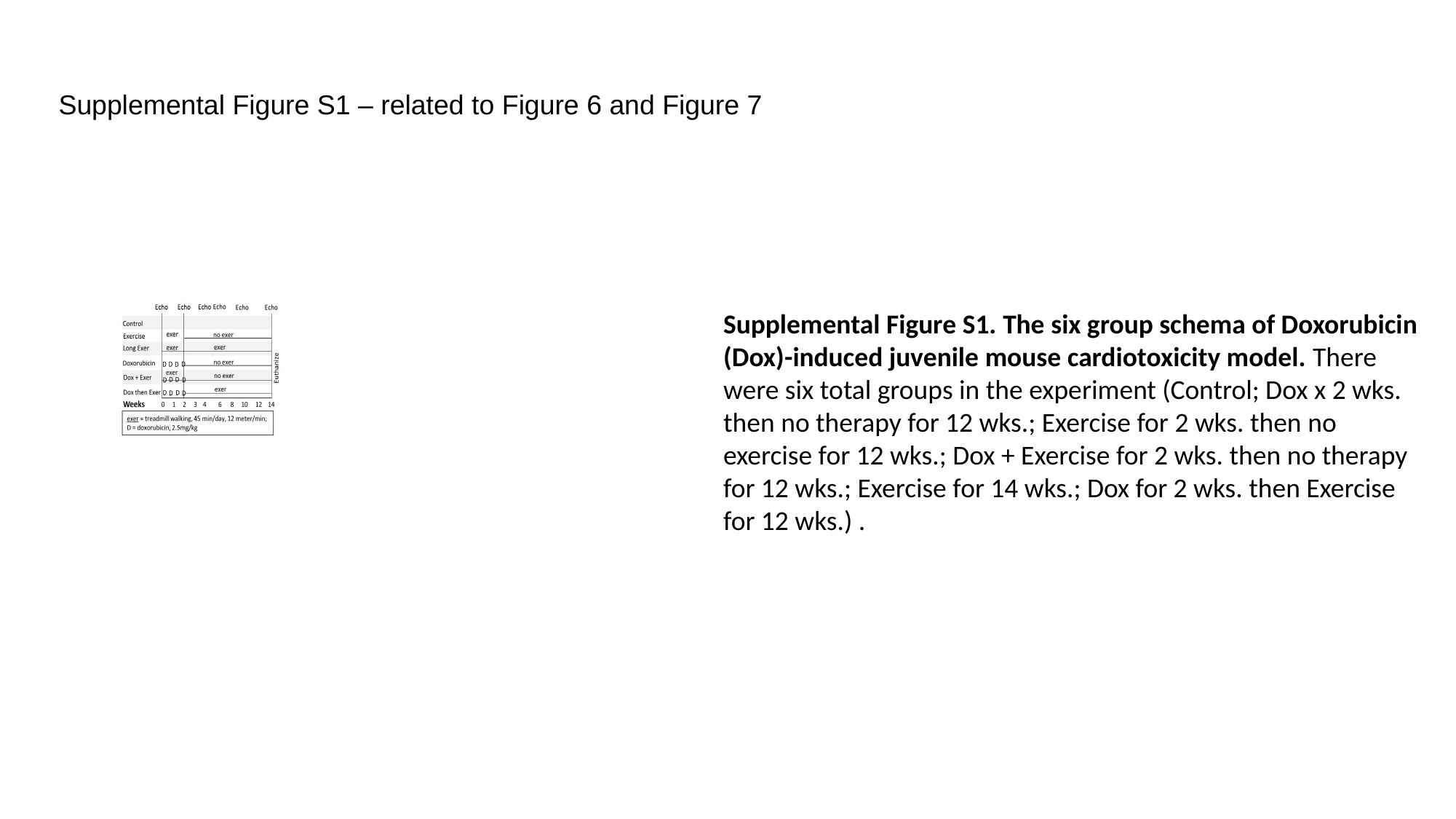

Supplemental Figure S1 – related to Figure 6 and Figure 7
Supplemental Figure S1. The six group schema of Doxorubicin (Dox)-induced juvenile mouse cardiotoxicity model. There were six total groups in the experiment (Control; Dox x 2 wks. then no therapy for 12 wks.; Exercise for 2 wks. then no exercise for 12 wks.; Dox + Exercise for 2 wks. then no therapy for 12 wks.; Exercise for 14 wks.; Dox for 2 wks. then Exercise for 12 wks.) .

## Slide 2
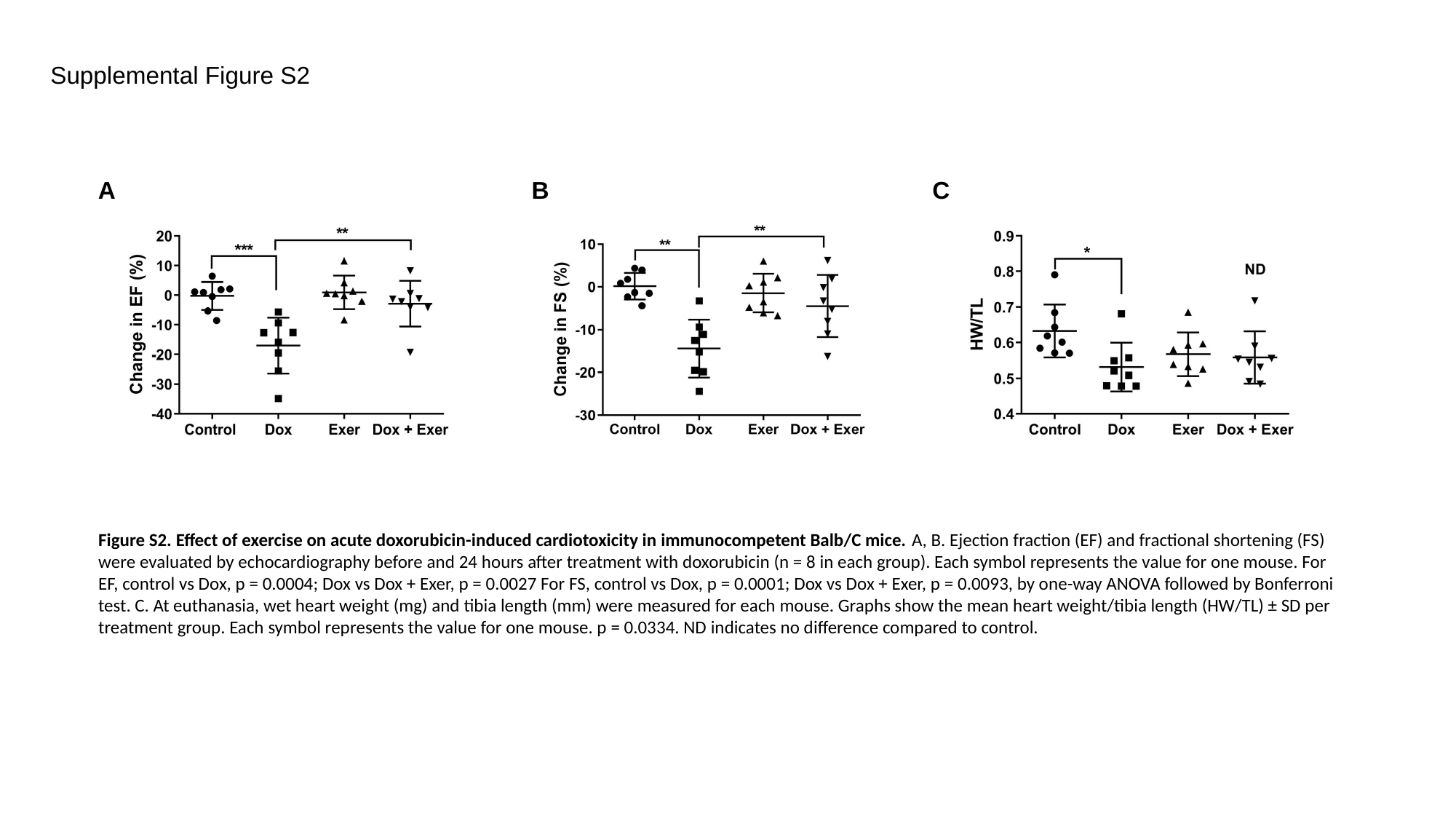

Supplemental Figure S2
A
B
C
Figure S2. Effect of exercise on acute doxorubicin-induced cardiotoxicity in immunocompetent Balb/C mice. A, B. Ejection fraction (EF) and fractional shortening (FS) were evaluated by echocardiography before and 24 hours after treatment with doxorubicin (n = 8 in each group). Each symbol represents the value for one mouse. For EF, control vs Dox, p = 0.0004; Dox vs Dox + Exer, p = 0.0027 For FS, control vs Dox, p = 0.0001; Dox vs Dox + Exer, p = 0.0093, by one-way ANOVA followed by Bonferroni test. C. At euthanasia, wet heart weight (mg) and tibia length (mm) were measured for each mouse. Graphs show the mean heart weight/tibia length (HW/TL) ± SD per treatment group. Each symbol represents the value for one mouse. p = 0.0334. ND indicates no difference compared to control.

## Slide 3
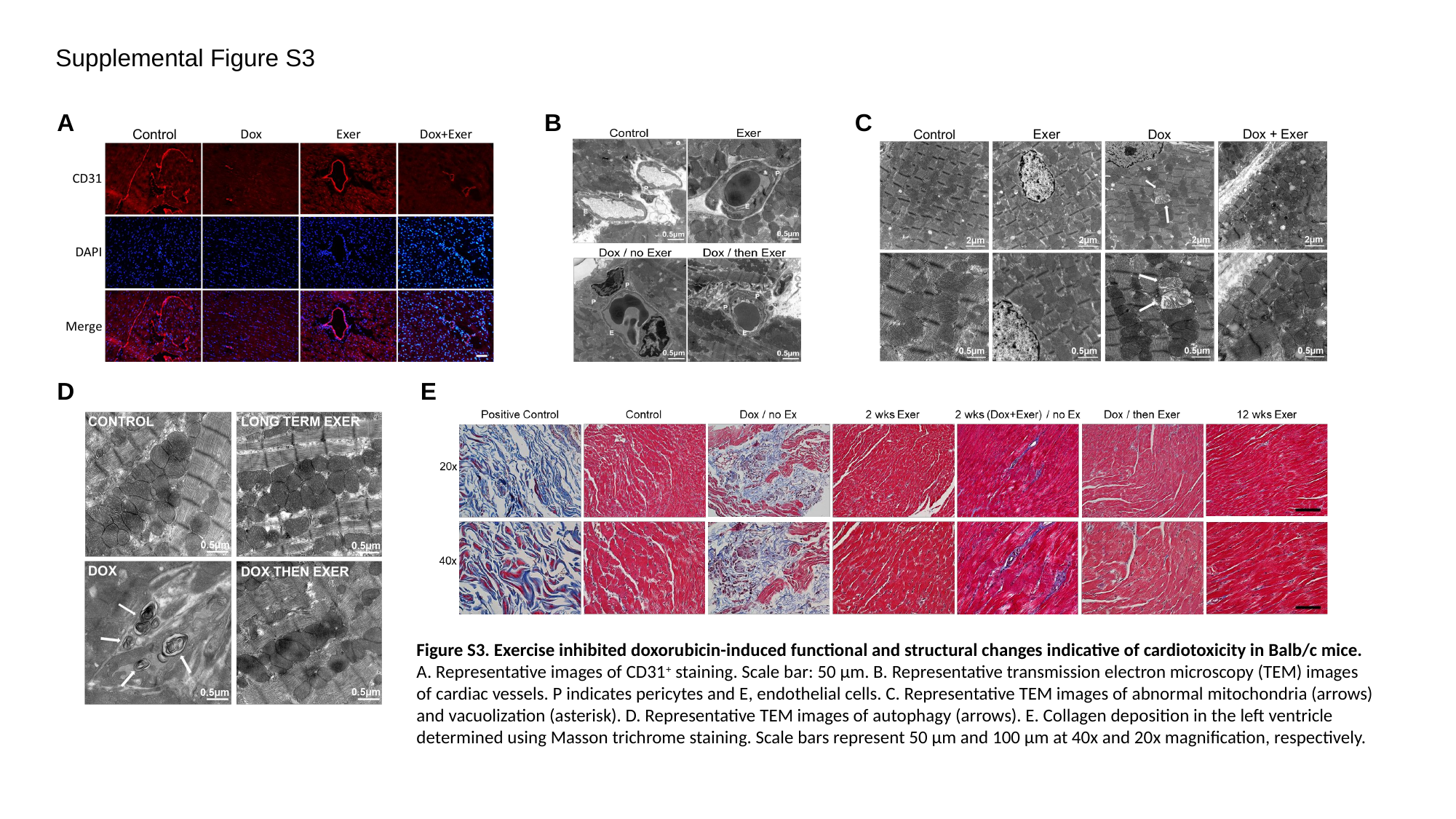

Supplemental Figure S3
A
B
C
D
E
Figure S3. Exercise inhibited doxorubicin-induced functional and structural changes indicative of cardiotoxicity in Balb/c mice. A. Representative images of CD31+ staining. Scale bar: 50 μm. B. Representative transmission electron microscopy (TEM) images of cardiac vessels. P indicates pericytes and E, endothelial cells. C. Representative TEM images of abnormal mitochondria (arrows) and vacuolization (asterisk). D. Representative TEM images of autophagy (arrows). E. Collagen deposition in the left ventricle determined using Masson trichrome staining. Scale bars represent 50 μm and 100 μm at 40x and 20x magnification, respectively.

## Slide 4
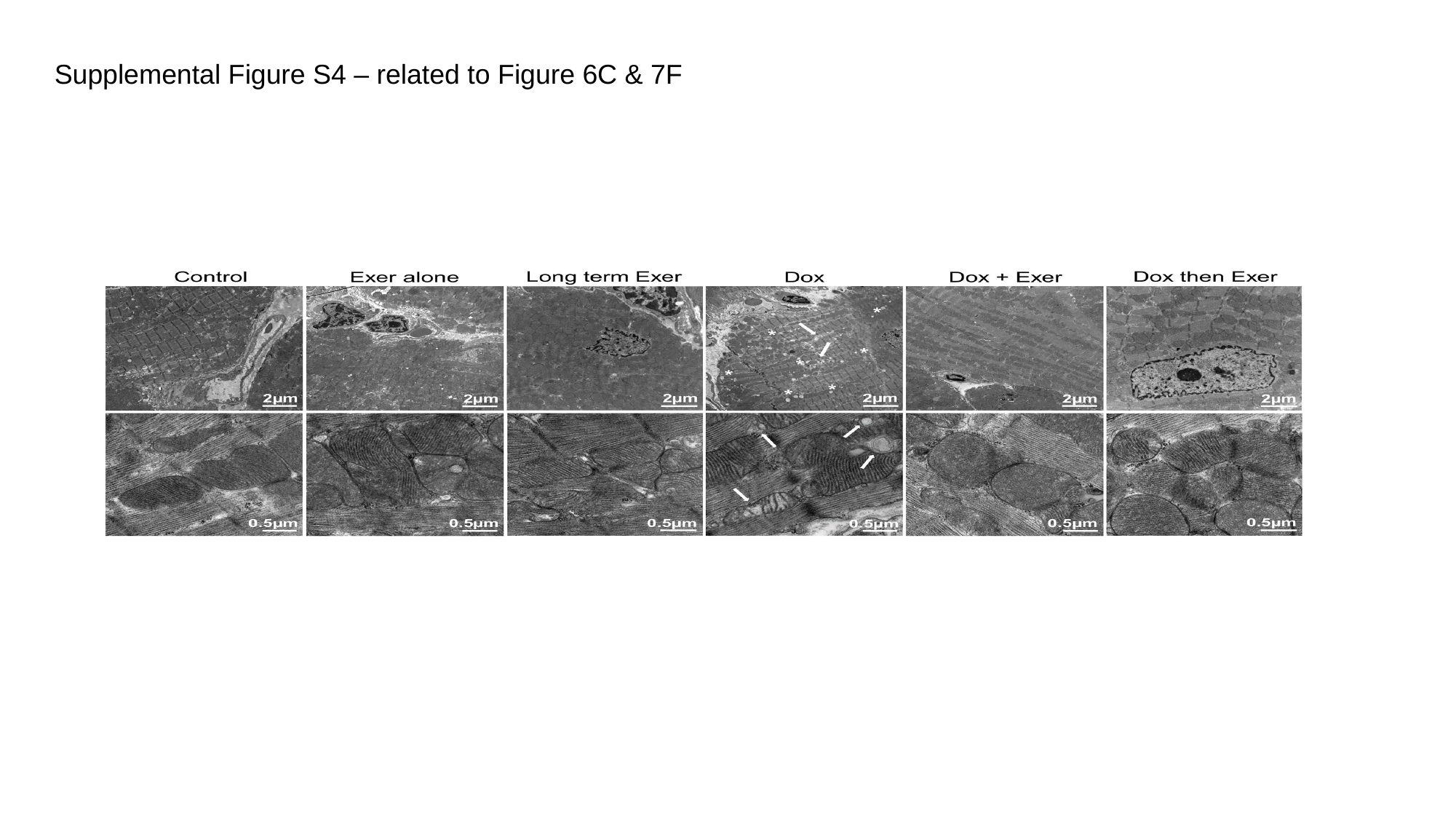

Supplemental Figure S4 – related to Figure 6C & 7F

## Slide 5
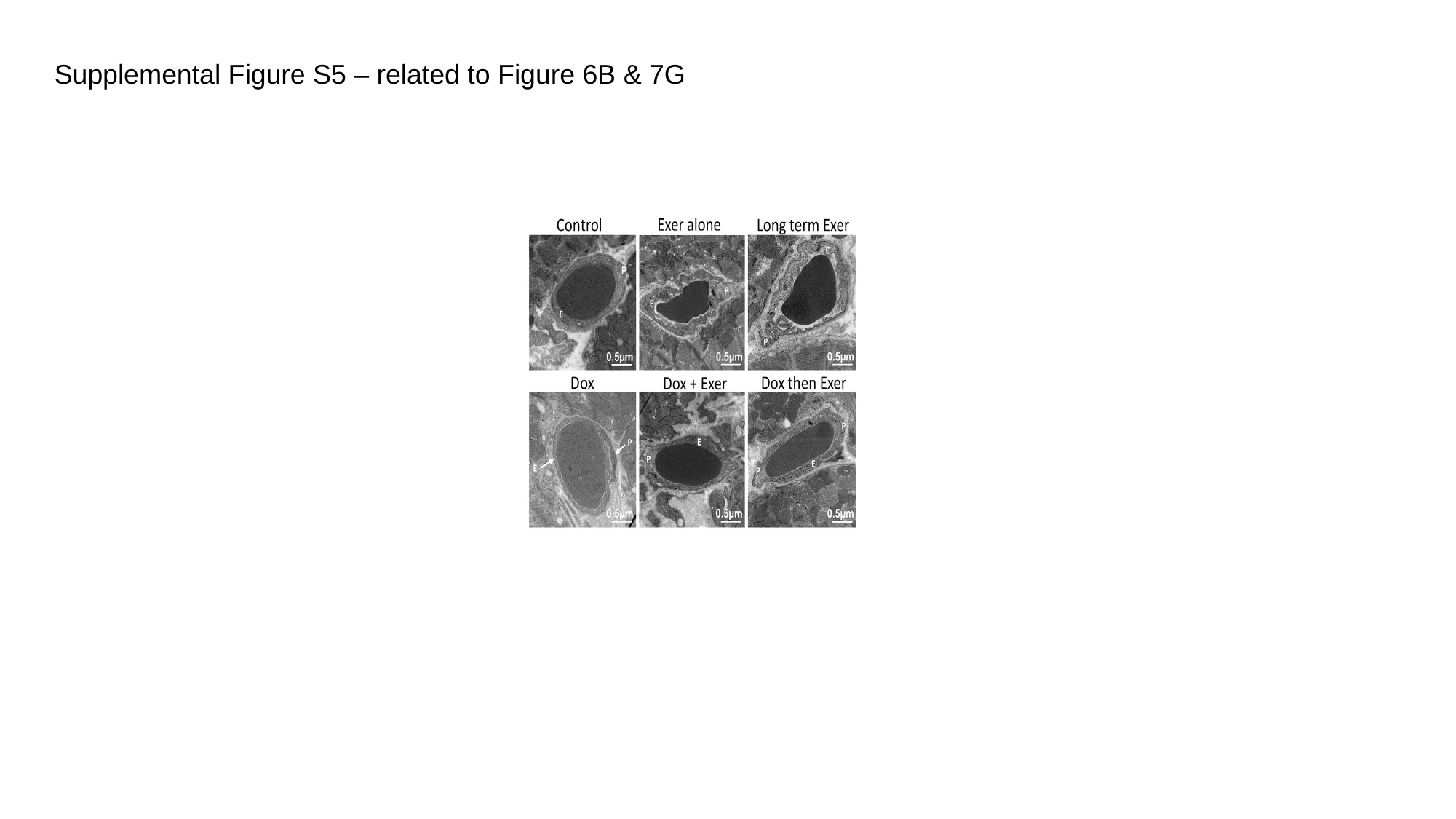

Supplemental Figure S5 – related to Figure 6B & 7G

## Slide 6
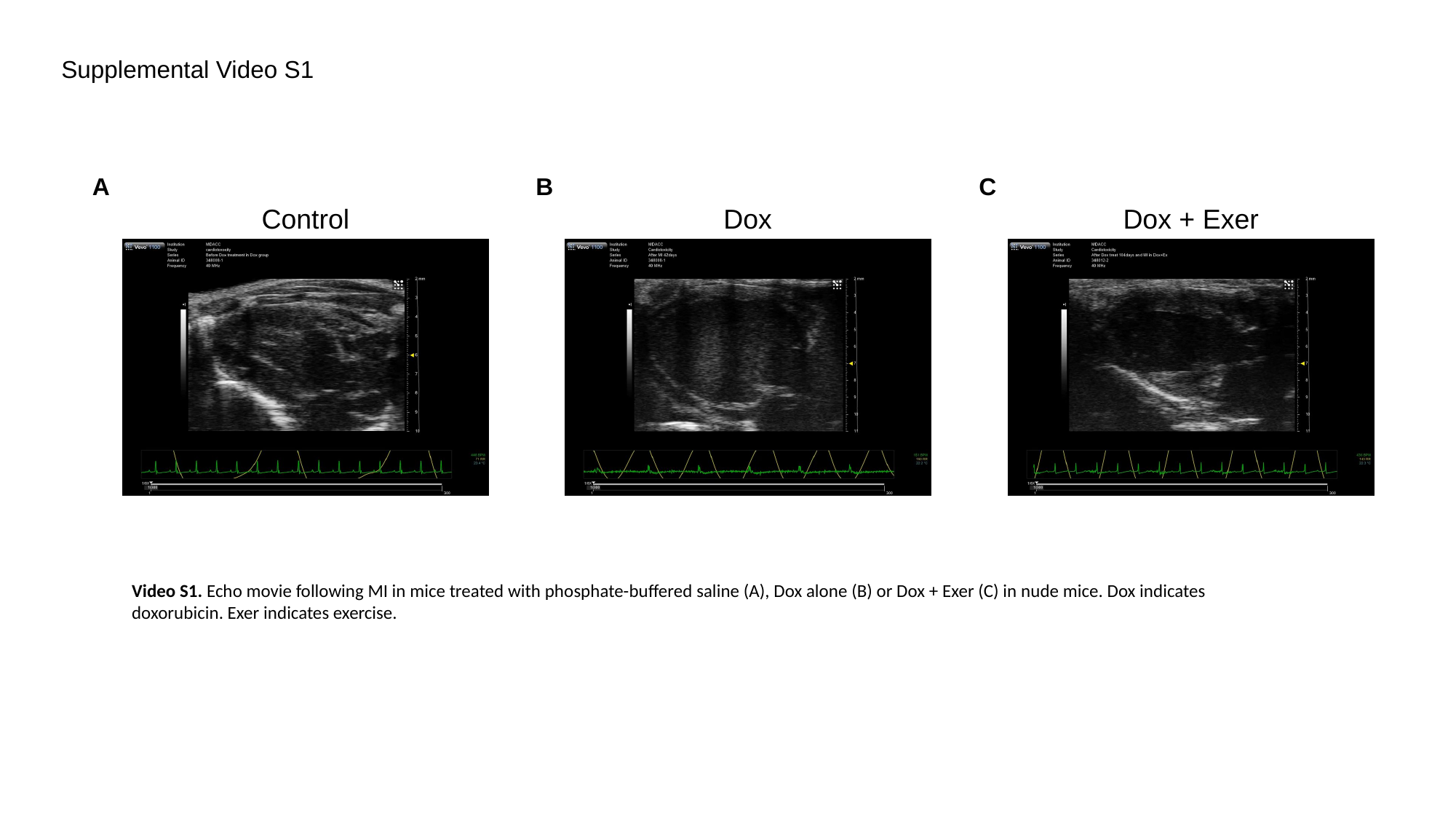

Supplemental Video S1
A
Control
B
Dox
C
Dox + Exer
Video S1. Echo movie following MI in mice treated with phosphate-buffered saline (A), Dox alone (B) or Dox + Exer (C) in nude mice. Dox indicates doxorubicin. Exer indicates exercise.
